# Lancemaside A, a major triterpene saponin of *Codonopsis lanceolata* enhances regulation of nitric oxide synthesis via eNOS activation

**DOI:** 10.1186/s12906-019-2516-6

**Published:** 2019-05-24

**Authors:** Young Seok Lee, HeeEun Kim, Jinhye Kim, Geun Hee Seol, Kwang-Won Lee

**Affiliations:** 1 Department of Biotechnology, College of Life Science and Biotechnology, Korea University, 212 CJ Food Safety Hall, Sungbuk-Gu, Anam-Ro 145, Seoul, 02841 South Korea; 2 Institute of Biomedical Science and Food Safety, Korea University, Seoul, 02841 South Korea; 3 Department of Basic Nursing Science, College of Nursing, Korea University, Seoul, 02841 South Korea

**Keywords:** *Codonopsis lanceolata*, Lancemaside A, Nitric oxide, Endothelial NO synthase, PI3K/Akt/eNOS signaling pathway, Hypertension

## Abstract

**Background:**

Many studies on the effect of saponin-rich *Codonopsis lanceolata* as a bioactive source for improving physical health have been performed. *C. lanceolata* contains triterpenoid saponins, including lancemasides. These saponins are known to be particularly involved in the regulation of blood pressure or hypertension. This study investigated whether lancemaside A (LA), a major triterpenoid saponin from *C. lanceolata*, regulates nitric oxide (NO) production via the activation of endothelial NO synthase (eNOS) in human umbilical vein endothelial cells.

**Methods:**

Upon separation with petroleum ether, ethyl acetate, and *n*-butanol, LA was found to be abundant in the *n*-butanol-soluble portion. For further purification of LA, HPLC was performed to collect fraction, and LA was identified using analysis of LC/MSMS and ^13^C-NMR values. In in vitro, the effects of LA on NO release mechanism in HUVECs were investigated by Griess assay, quantitative real-time reverse-transcription PCR, and Western blotting.

**Results:**

Our results showed that NO production was efficiently improved by treatment with LA in a dose-dependent manner. In addition, the LA treatment resulted in extensive recovery of the NO production suppressed by the eNOS inhibitor, L-NAME, compared with that in the control group. Additionally, the level of eNOS mRNA was increased by this treatment in a dose-dependent manner. These results suggested that LA is an inducer of NO synthesis via eNOS mRNA expression. Also, the study indicated that LA is involved in activating the PI3K/Akt/eNOS signaling pathway.

**Conclusion:**

These results suggested that LA is an inducer of NO synthesis via eNOS mRNA expression. Also, the study indicated that LA is involved in activating the PI3K/Akt/eNOS signaling pathway. These findings suggest the value of using LA as a component of functional foods and natural pharmaceuticals.

**Electronic supplementary material:**

The online version of this article (10.1186/s12906-019-2516-6) contains supplementary material, which is available to authorized users.

## Background

*Codonopsis lanceolata* is a natural ingredient, which is edible raw or cooked, and has also been used as a traditional medicine in East Asia for the treatment of inflammatory disorders such as cough, bronchial asthma, tonsillitis, and pharyngitis [1, 2]. *C. lanceolata* extract has also been reported to protect the liver damage [3, 4], counter obesity [5], induce memory development [6, 7], and inhibit tumors [8]. *C. lanceolata* contains various phytochemical components, including many different triterpenoid saponins such as lancemaside A–C, E, and G, foetidissimoside A, and aster saponin Hb [9]. Although the effects of other saponin compounds from *Astragalus corniculatus* [10], *Panax notoginseng* [11], and soybean [12] are widely known, the effects of saponin-rich *C. lanceolata* remain to be fully explained and evaluated in terms of other disease applications.

Recently, we reported that *C. lanceolata* extract is effective in preventing hypertension and reducing systolic blood pressure (SBP) in rats [13]. The treatment of hypertentive rats with both 200 mg and 400 mg of *C. lanceolata* extract per kg body weight significantly reduced SBP compared with the hypertentive vehicle, whereas the plant extract did not decrease SBP in normotensive rats. We hypothesized that lancemaside A (LA) occurring in this plant contributes to these hypotensive effects, because LA is a major triterpenoid saponin contained in *C. lanceolata*.

Lancemaside A is reported to enhance anti-inflammatory function by blocking IKK/NF-κB activation on RAW 264.7 and U937 cells [14] and potently ameliorates colitis via TLR-linked NF-κB activation in mice [15]. In addition, LA was reported to suppress microglial activation, which plays an important role in neurodegenerative diseases via regulation of the JNK signaling pathway [16]. Moreover, LA and its metabolite, echinocystic acid, improved scopolamine-induced memory and learning deficits in mice by inhibiting acetylcholinesterase activity and inducing brain-derived neurotrophic factor and phosphorylated cAMP response element binding protein (p-CREB) expression.

Endothelial nitric oxide synthase (eNOS), which synthesizes a nanomolar amount of nitric oxide (NO), is an isozyme of NOS; other NOS isozymes include neuronal NOS (nNOS), inducible NOS (iNOS), and bacterial NOS (bNOS) [17]. eNOS phosphorylation is mediated through PPAR-γ-eNOS, oxidative stress, and Rho-kinase pathway [18], angiopoietin-related growth factor [19], VEGF-mediated focal adhesion kinase (FAK) phosphorylation in hypoxia [20], or the phosphatidylinositol-3-kinase (PI3K)/protein kinase B (PKB, Akt) signaling pathway to regulate NO synthesis [21]. NO, which plays an important role in modulating blood pressure, is formed from L-arginine by eNOS phosphorylation [22]. Synthesized NO stimulates soluble guanylyl cyclase (sGC), which is a receptor for NO in smooth muscle cells (SMC), thereby converting guanosine triphosphate (GTP) to cyclic guanosine monophosphate (cGMP) [23]. Activation of sGC leads to vascular relaxation via NO-cGMP signaling [24]. These endothelial functional effects of saponin from ginseng, such as ginsenoside-Rg1, on NO production via the PI3K/Akt/eNOS signaling pathway in HUVECs are well known [25, 26]. However, no study has yet reported the effects of LA on NO synthesis through eNOS phosphorylation.

To bridge this research gap, LA was isolated from the ethanol extract of *C. lanceolata* and identified by several instrumental approaches. In this study, efforts were then made to evaluate its effect on NO production via eNOS activation.

## Methods

### Chemicals and materials

Materials were purchased respectively as follows: EGM-2 medium kit from Lonza Cambrex (Nottingham, UK), enhanced chemiluminescence (ECL) reagent from AbClon (Seoul, South Korea), Griess reagent from Promega Co. (WI, USA), LeGene Premium Express 1st Strand cDNA Synthesis System from LeGene Biosciences (CA, USA), polyvinylidene fluoride (PVDF) membranes from Millipore (MA, USA), pyridine-*d5* from Cambridge Isotope Laboratories Inc. (MA, USA), RNAiso PLUS from TAKARA Korea Biomedical Co. (Seoul, South Korea), thin-layer chromatography (TLC) silica gel 60 F_254_ from Merck (Darmstadt, Germany), and TOPreal™ qPCR 2× PreMIX SYBR green from Enzynomics (Seoul, South Korea). N(G)-nitro-L-arginine methyl ester (L-NAME), fetal bovine serum (FBS), and silica gel resin were purchased from Sigma-Aldrich (MO, USA). All other chemicals were of ultra-pure grade. The primary antibodies (eNOS, phospho-eNOS Ser^1177^, Akt, phospho-Akt Thr^308^, and GAPDH) and horseradish peroxidase (HRP)-conjugated secondary antibodies (anti-rabbit and anti-mouse) were obtained from Merckmillipore (CA, USA). All other chemicals were of ultra-pure grade.

### Separation of LA from *C. lanceolata*

#### Plant materials

The edible rhizomes of *C. lanceolata* were identified and obtained from PANAX KOREA Co., Ltd. (Gangwon-do, South Korea). The voucher specimen (KUH-359) was deposited at Korea University Herbarium. The fresh rhizomes were washed and sliced, and then the sliced rhizomes were immediately dried in a freeze-dryer. The dried *C. lanceolata* was finely ground in a mortar and kept refrigerated at 4 °C.

#### Extraction, isolation, and isolation of LA from *C. lanceolata*

The parched rhizomes of *C. lanceolata* (100 g) were extracted with 55% ethanol at 60 °C for 4.5 h using a reflux condenser and then cooled. The undissolved remains were filtrated using Whatman qualitative filter paper 2 (Whatman Inc., Clifton, NJ, USA). The filtrate was concentrated using a rotary vacuum evaporator (N-1000S; EYELA, Tokyo, Japan) and then lyophilized to yield 46.31 g of powder. Our group reported that LC/MS analysis of the compounds from this ethanol extract of *C. lanceolata* contain lancemaside A, B, C, E, and G, foetidissimoside A, and aster saponin Hb (Han et al. 2018). For further fractionation of the dried *C. lanceolata* extract, the extract was resuspended in H_2_O and then successively extracted with petroleum ether, ethyl acetate, and *n*-butanol. The *n*-butanol-soluble portion (7.79 g) was applied to silica gel open column chromatography and eluted with a gradient of increasing methanol in dichloromethane (DCM). Finally, the active fraction of DCM and methanol (60:40, v/v) (0.49 g) was further separated using the Agilent Technologies 1200 Series HPLC system. The separation was performed using a YMC-Triart C_18_ column (5 μm, 4.6 × 250 mm). HPLC separation was performed with a flow rate of 1 mL/min (injection volume, 20 μL), with a linear gradient of solvent mixtures of 0.05% trifluoroacetic acid (TFA) in H_2_O (solvent A) and CH_3_CN (acetonitrile, ACN) (solvent B) increasing ACN to 100% for 60 min. Fractions were collected by a fraction collector (Model 2110; Bio-Rad, USA) at 1-min intervals. The eluted fraction was analyzed using TLC, and LA was isolated (0.22 g) (Additional file 1: Figure S1).

#### Thin-layer chromatography

To select the active fractions that contain LA during the isolation and purification processes, TLC was used. Each HPLC fraction was loaded (100 μg/lane) on a TLC plate coated with silica gel (TLC silica gel 60 F_254_; Merck, Darmstadt, Germany). The plate was introduced into a saturated TLC chamber consisting of a mobile phase (chloroform–methanol–water 65:35:10, v/v/v) for 30 min at room temperature. Color formation was performed by 10% sulfuric acid, followed by heating to 110 °C on a hot plate.

#### Liquid chromatography-tandem mass spectrometry analysis (LC-MS/MS)

Stock samples (1 mg/mL) of LA for LC-MS/MS were prepared by dissolving 20 μg/mL LA in 50% methanol (MeOH). The LC-MS/MS analysis was performed by coupling a Nexera X2 HPLC system (Shimadzu, Kyoto, Japan) and an LCMS-8050 mass spectrometer (Shimadzu, Kyoto, Japan) as an electrospray ionization (ESI) source. The mass spectrometric conditions and the optimized multiple reaction monitoring parameters are shown in Additional file 2: Table S1.

#### ^13^C-nuclear magnetic resonance (NMR) analysis

An NMR experiment was performed to determine the ^13^C-NMR spectral patterns of LA in pyridine-*d*_5_ (15 mg/mL). Specifically, the analysis was conducted using a liquid-phase cryoporometry NMR system (Avance-600; Bruker, Germany) operating at 600 MHz. To compare the observed ^13^C-NMR spectrum of LA, ^13^C-NMR data were obtained from the literature [27–29]. The chemical shifts are shown as δ-values (ppm).

### Cell culture

HUVECs were obtained from Lonza Cambrex (Seoul, South Korea) and cultured in endothelial growth medium-2 (EGM-2) supplemented with 2% FBS under the condition of a humidified atmosphere at 37 °C with 5% CO_2_. Cells from the third to the sixth passage in an active condition were used in this experiment. The cells were treated with various concentrations of LA diluted in EGM-2 (0.5, 1, 5, 10, and 15 μM). For an inhibitor assay with eNOS, HUVECs were treated with an eNOS-specific inhibitor, L-NAME (25 μM) for 24 h before the treatment of LA.

### Measurement of NO production

The HUVECs were cultured on 6-well plates at cell density of 5 × 10^5^ cells/well. To evaluate NO production, the HUVEC cells were treated with LA (0.5, 1, 5, 10, and 15 μM) for 24 h and an eNOS-specific inhibitor, L-NAME (25 μM) for 24 h before the treatment of LA (15 μM) for 24 h, following the medium was collected to measure the amount of NO released by the cells using the Griess assay. The supernatant medium (100 μL) in a 96-well plate was mixed with an equal volume of Griess reagent (1% sulfanilamide in 5% H_3_PO_4_ and 0.1% N-[1-naphthyl]-ethylenediamine dihydrochloride) and incubated for 10 min. Subsequently, OD was measured spectrophotometrically at 540 nm using a multi-plate spectrometer.

### Quantitative real-time reverse-transcription PCR

mRNA expression was measured by quantitative real-time reverse-transcription polymerase chain reaction (qRT-PCR). Total RNA isolation, cDNA synthesis, and mRNA expression were performed using RNAiso PLUS (TAKARA Korea Biomedical Co., Seoul, South Korea), LeGene Premium Express 1st Strand cDNA Synthesis System (LeGene Biosciences, CA, USA), and TOPreal™ qPCR 2X PreMIX SYBR Green (Enzynomics, Seoul, South Korea), respectively following the manufacturer’s instructions. The cycle conditions of an iQ5 thermal cycler (Bio-Rad, CA, USA) were as follows: 3 min at 95 °C, followed by 30 cycles of incubation at 95 °C for 3 s, 61 °C for 50 s, and 72 °C for 1 min. The level of eNOS mRNA expression was normalized to the corresponding value for GAPDH mRNA expression. The sequences of eNOS and GAPDH primers for qRT-PCR analysis were “GGA CGGAGCTGGCTGC” and “GCGTATGCGGCTTGTCAC”, and these primers accession number in Entrez Nucleotide database is AF400594.

### Western blotting assay

For elucidation of the effect of LA on the phosphorylation of eNOS, HUVECs were treated with LA (15 μM) for 20 min and L-LAME (25 μM) for 24 h. In addition, for investigation of the function of LA in activation of the Pi3K/Akt/eNOS signaling pathway, HUVEC were treated with LA and L-LAME for 20 min and co-incubated with or without a PI3K inhibitor, LY294002 (25 μM), and an Akt phosphorylation inhibitor, A6730 (5 μM), for 15 h. After the cells had been subjected to different treatments, all cells were rinsed twice with ice-cold phosphate-buffered saline (pH 7.2) and lysed at 4 °C in RIPA lysis buffer containing proteases (1:1000) and phosphatase inhibitors (1:100). After the cell lysates had been centrifuged at 13,000 rpm for 20 min at 4 °C, the supernatant was used as the cellular protein sample. The protein sample (20 μg/lane) was separated by electrophoresing in 7.5% gel and transferred onto PVDF membrane sheets. The sheets were blocked with 5% nonfat dry skim milk or bovine serum albumin in TBST [20 mM Tris-HCl (pH 7.4), 500 mM NaCl, and 0.1% Tween-20] at room temperature for 1 h. Then, the sheets were bound with specific primary antibodies at 4 °C overnight, targeting eNOS, phospho-eNOS Ser^1177^, phospho-Akt Thr^308^ (dilution 1:1000), Akt (dilution 1:1500), and GAPDH (dilution 1:2000). After rinsing five times with TBST for 30 min, the sheets were probed with HRP-conjugated secondary antibodies (dilutions: anti-rabbit, 1:2000; anti-mouse, 1:4000) at room temperature for 45 min and rinsed five times with TBST for 30 min. The immunoreactive protein–antibody complexes were visualized using an ECL reagent and scanned by a luminescence image analyzer (LAS-4000; Fuji Film, Tokyo, Japan). The bands were normalized to the GAPDH levels using Image J software (National Institutes of Health, MD, USA).

### Statistical analysis

All results are expressed as mean ± standard deviation of three independent experiments conducted in triplicate. Different letters indicate significant differences at *P < 0.05, as determined by Duncan’s multiple-range test. All statistical analyses were performed using SAS version 9.4 (SAS Institute, NC, USA).

## Results

### Isolation and detection of LA from *C. lanceolata*

In previous reports on the separation of lancemaside A, *C. lanceolata* was extracted with methanol to separate the material [14, 30]. After their continuous extraction with organic solvents, yields of lancemaside A were 0.11 and 0.13%. In this study, we sought to extract *C. lanceolata* with an environmentally friendly solvent, ethanol and to report in detail on isolation and identification of lancemaside A for increasing the yield of compared with that from previous methods.

Analysis of the eluted fraction was performed using TLC (Additional file 1: Figure S1a). After TLC plates spotted with the fraction were developed in a developing solvent mixture of chloroform/MeOH/H_2_O (65:35:10) and dried, the plates containing LA were effectively detected as dark-brown spot, after spraying with 10% sulfuric acid. Upon separation with petroleum ether, ethyl acetate, and *n*-butanol, LA was found to be abundant in the *n*-butanol-soluble portion (top panel of Additional file 1: Figure S1b). Next, the portion was fractionated into 11 fractions (open column fractions 1–11) by silica gel open column chromatography using a DCM–methanol solvent gradient. As shown in the middle panel of Additional file 1: Figure S1b, LA was mainly identified in open column fraction #5 (DCM–methanol 60:40, v/v). For further purification of LA, HPLC was performed to collect fractions in 1-min intervals for 60 min (HPLC fractions 1–60). The bottom panel of Additional file 1: Figure S1b shows that purified LA was obtained from HPLC fraction. The yield of lancemaside A was 0.22%.

### Identification of LA from *C. lanceolata*

**Fig. 1:**
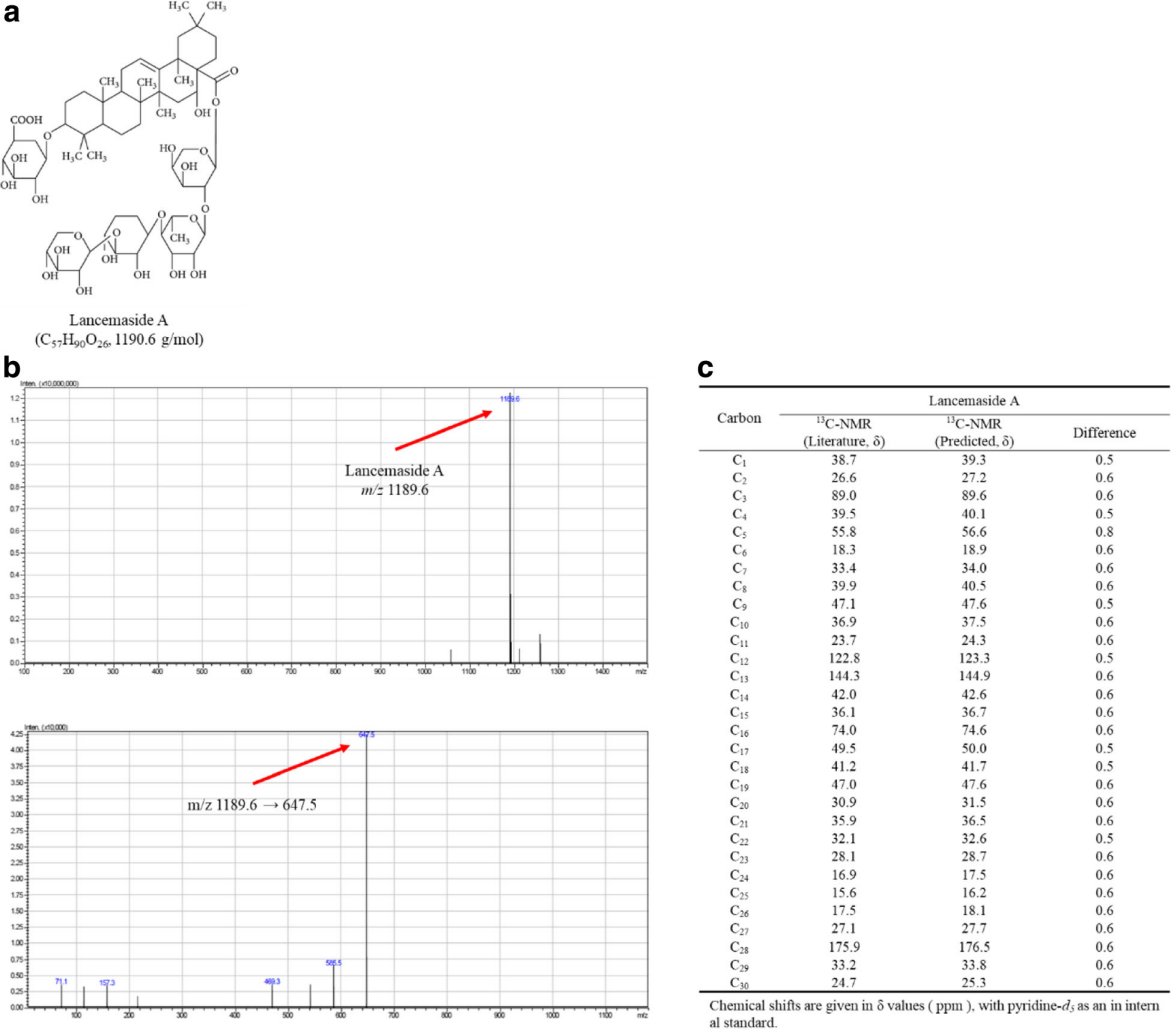
Identification of LA from *C. lanceolata*. **a** Structure of LA (**b**) Mass spectra of precursor ion of LA and their fragmentation product ion were obtained in ESI negative-ion detection mode ([M-H]^−^). In the mass spectrum, LA showed the ions to be at m/z 1189.6 (top panel). The tandem mass spectrum of the relative fragmentation product ions was monitored at m/z 1189.6 → 647.5 for LA (bottom panel). The product ion at m/z 647.5 was produced by loss of a tetrasaccharide unit (arabinose, rhamnose, and two xyloses). **c** For analysis of the correlation of the ^13^C-NMR values (δ, ppm) of LA, the ^13^C-NMR data of LA were obtained from the literature. To validate the ^13^C-NMR data of LA, the chemical shifts of LA were observed and compared with the literature data

LA (R_t_ of approximately 3.11 min) in ESI-MS with negative-ion mode gave a deprotonated molecule ([M-H]^−^ precursor ion) at *m/z* 1189.6, with the structure of the compound indicating a molecular weight of 1190.6 and the formula C_57_H_90_O_26_ (Fig. 1a, top panel of Fig. 1b). The molecular ion [M-H]^−^ produced three peaks of product ions at *m/z* 469.2, 585.3, and 647.5 in MS/MS (bottom panel of Fig. 1b). Among these ion peaks, the major fragmentation peak of LA was at *m/z* 647.5, which was identified as LA [31]. In the MS/MS spectrum of the [M-H]^−^ ion at *m/z* 1189.6, the *m/z* 647.5 ion ([M-H]^−^) was produced by the neutral loss of a tetrasaccharide unit (arabinose, rhamnose, and two xyloses) [32]. For analysis of the correlation of the ^13^C-NMR values (δ, ppm) of LA (Fig. 1c), the ^13^C-NMR data of LA were obtained from the literature [27, 31, 33]. To validate the ^13^C-NMR data of LA, the chemical shifts of LA were observed and compared with the literature data.

### Effects of LA on NO synthesis

**Fig. 2:**
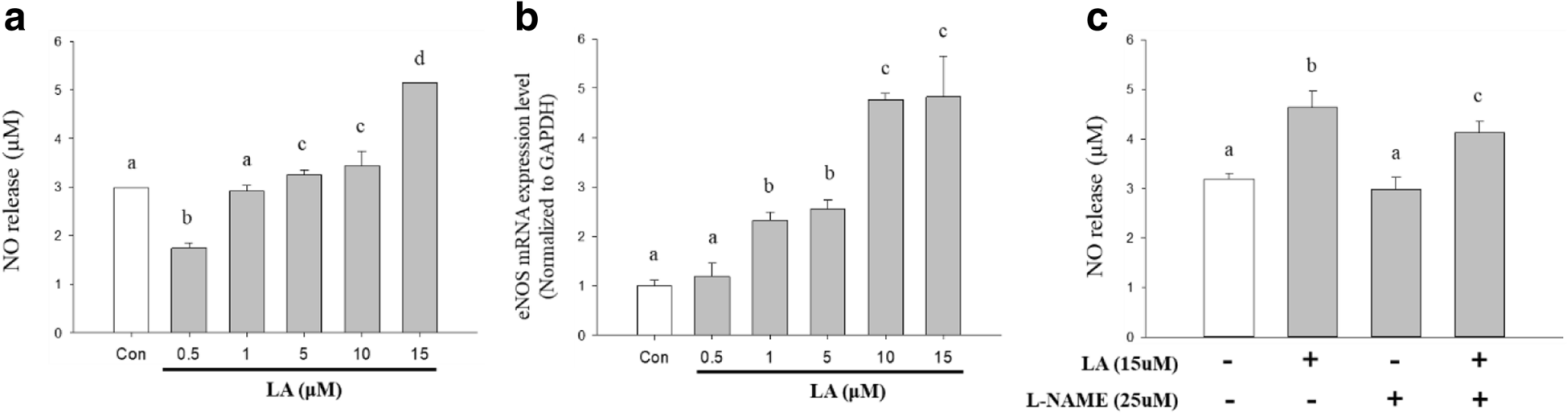
The effects of LA on nitric oxide synthesis in HUVECs. **a** HUVECs were treated with various concentrations of LA (0.5, 1, 5, 10, and 15 μM) for 24 h, followed by the Griess assay. Con - non-treated negative control (**b**) The expression of eNOS mRNA levels in HUVECs, the cells were treated with various concentrations of LA (0.5, 1, 5, 10, and 15 μM) for 24 h, followed by the measurement of eNOS mRNA expression levels using qRT-PCR. These data are normalized by the mRNA expression levels of GAPDH. **c** Measurement of the activity of LA against the L-N(**g**)-nitro-L-arginine methyl ester (L-NAME)-induced decrease of nitric oxide (NO) production. The cells were treated with 15 μM LA for 24 h, after which they were treated with 25 μM L-NAME for 24 h, followed by the Griess assay. Different letters indicate a statistically significant difference between each group at *P < 0.05, as determined by Duncan’s multiple-range test

Compared with that in the control group, treatment with various concentrations of LA significantly increased NO production in a dose-dependent manner except the lowest LA level (0.5 μM) showing a NO’s releasing inferior to the control group (Fig. 2a). As shown in Fig. 2b, treatment of HUVECs with LA significantly increased eNOS mRNA expression in a dose-dependent manner. The results showed that the reduced level of NO production in L-NAME-treated cells was recovered upon treatment with LA (Fig. 2c). Since 15 μM LA did not affect cell viability (above 85%) and was effective for NO production in the cells, this concentration was used in further experiments.

### Effects of LA on the phosphorylation of eNOS and Akt

**Fig. 3:**
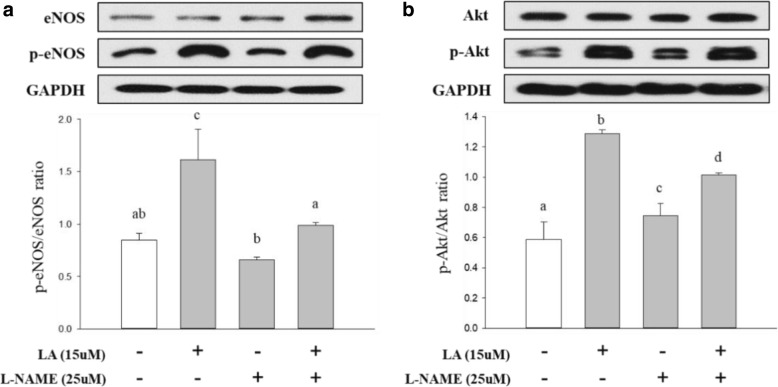
Effects of LA on endothelial NO synthase (eNOS) phosphorylation in HUVECs. HUVECs treated with LA (15 μM) for 20 min and co-incubated with or without L-NAME (25 μM) for 24 h, after which total lysates of cells were obtained by RIPA buffer containing protease inhibitors and phosphatase inhibitors. **a** LA treatment of phospho-eNOS and total eNOS, and (**b**) LA treatment of phospho-Akt and total Akt from HUVECs stimulated with LA. Different letters indicate a statistically significant difference between each group at *P < 0.05, as determined by Duncan’s multiple-range test

After treatment with LA, as shown in Fig. 3a, compared with that in the control group, this treatment increased eNOS phosphorylation, whereas this phosphorylation was restrained by treatment with L-NAME. Moreover, eNOS phosphorylation, which was reduced by treatment with L-NAME, was partially recovered by treatment with LA. Akt phosphorylation was analyzed using Western blotting. Figure 3b shows that LA, but not L-NAME, also contributed to Akt phosphorylation.

### Effect of LA on stimulation of the PI3K/Akt/eNOS signaling pathway

**Fig. 4:**
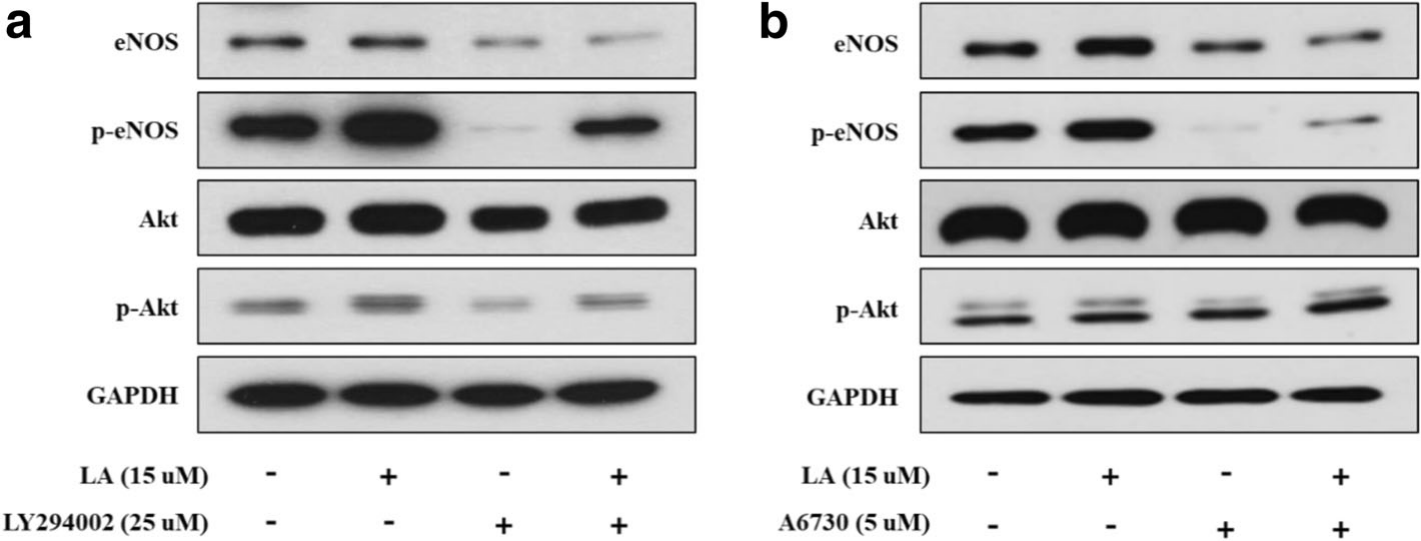
Effects of LA on eNOS phosphorylation via the PI3K/Akt signaling pathway in HUVECs. HUVECs treated with LA (15 μM) for 20 min and co-incubated with or without PI3K inhibitor (LY294002, 25 μM) and an Akt phosphorylation inhibitor (A6730, 5 μM) for 15 h. **a** Phosphorylation of eNOS and Akt was inhibited by LY294002; however, it was enhanced by treatment with LA, and (**b**) A6730 also suppressed the phosphorylation of eNOS and Akt, but their phosphorylation was improved by LA treatment

Western blotting was performed to investigate the protein levels of Akt, p-Akt, eNOS, and p-eNOS (Fig. 4). As shown in Fig. 4a, LA significantly increased p-eNOS and p-Akt inhibited by LY294002. Likewise, LA substantially improved and reversed p-eNOS and p-Akt blocked by A6730 (Fig. 4b). Moreover, LY294002 and A6730 can inhibit eNOS phosphorylation.

## Discussion

The method using TLC developing solvent mixture of chloroform/MeOH/H_2_0 (65:35:10) was very effective for screening those containing LA among fraction samples, after which the detection was easily confirmed with 10% sulfuric acid spray on TLC plate.

Many studies on the effect of saponin-rich *C. lanceolata* as a bioactive source for improving physical health have been performed [3, 4, 34–39]. These saponins are known to be particularly involved in the regulation of blood pressure or hypertension. The bioactivities of LA which is a saponin, isolated from *C. lanceolata* remain to be sufficiently elucidated [40]. In this study, NO synthesis with LA treatment in human umbilical vein endothelial cells, HUVECs was investigated. The HUVECs have been widely used as a laboratory in vitro model system for studying endothelial cell functions and responses to stress [41]. We made efforts to identify LA from the ethanol extract of *C. lanceolata* by several instrumental approaches and to evaluate its effect on NO production via eNOS activation.

Endothelial NOS is one of four isoforms of NOS, which also include iNOS, nNOS, and bNOS [42, 43]. NO synthesis, which is regulated by eNOS in endothelial cells, is closely connected to vasodilation related to blood pressure [44]. It has been reported that NO released by HUVECs activates sGC, which is a NO receptor in SMC, thereby converting GTP to cGMP, and this activation can lead to vascular relaxation [24, 45]. To elucidate the effect of LA on the phosphorylation of eNOS, which is a protein involved in NO production, HUVECs were treated with LA (15 μM) and L-NAME (25 μM). Our results showed that NO production was efficiently improved by treatment with LA in a dose-dependent manner. Furthermore, as the effect of LA against L-NAME-inhibited NO production, the LA treatment resulted in extensive recovery of the NO production suppressed by the eNOS inhibitor, L-NAME, compared with that in the control group. In addition, this treatment increased the level of eNOS mRNA in a dose-dependent manner. These results suggested that LA is an inducer of NO synthesis via eNOS mRNA expression. The phospho-eNOS in the vascular endothelium aggressively induces NO generation, leading to the regulation of vascular tone and cellular proliferation [46]. eNOS phosphorylation progressed remarkably compared with that in the untreated group and the group treated with the eNOS inhibitor, L-NAME (Fig. 3). Furthermore, protein kinase B (PKB/Akt) is well known to elevate eNOS phosphorylation [47]. As determination of eNOS activation, LA significantly increased and recovered p-eNOS and p-Akt levels although PI3K/Akt/eNOS signaling pathway inhibition was induced by LY294002 and A6730 (Fig. 4). These results demonstrated that Akt phosphorylation is involved in eNOS phosphorylation related to NO production and that LA is involved in activating the PI3K/Akt/eNOS signaling pathway. On the other hand, it would be more interesting to study further structure-activity relationship of LA.

## Conclusions

This study demonstrated that LA, a saponin from C. lanceolate, enhances endothelial cell function, such as NO synthesis, via eNOS activation in the PI3K/Akt/eNOS signaling pathway, suggesting the value of using LA as a component of functional foods and natural pharmaceuticals.

## Additional files

Additional file 1: **Figure S1.** Determination of lancemaside A (LA) from *C. lanceolata*. (a) Scheme showing the isolation of LA from *C. lanceolata.* (b) Thin-layer chromatography, TLC analysis was performed to identify LA in the *n*-butanol-soluble portion (top panel). Open column was packed with the silica gel fractions (3–7) eluted by a dichloromethane-methanol solvent gradient (middle panel) and HPLC fractions (27–29) were separated by a linear gradient of solvent mixtures of 0.05% trifluoroacetic acid in H_2_O (TFA, solvent A) and acetonitrile (solvent B) (bottom panel) (DOCX 399 kb)

Additional file 2: **Table S1.** Liquid chromatography-tandem mass spectrometry (LC-MS/MS) conditions for the determination of lancemaside A (LA) (A) and summary of MS & MS/MS chromatogram spectrum in negative ion mode of LA (B) (DOCX 25 kb)

## Abbreviations

ACN: Acetonitrile
bNOS: bacterial NO synthase
cGMP: cyclic guanosine monophosphate
CREB: cAMP responsive element binding protein
DCM: Dichloromethane
ECL: Enhanced chemiluminescence
EGM-2: Endothelial growth medium-2
eNOS: endothelial NOS
FAK: Focal adhesion kinase
FBS: Fetal bovine serum
GTP: Guanosine triphosphate
HRP: Horseradish peroxidase
iNOS: inducible NOS
LA: Lancemaside A
L-NAME: L-N(G)-nitro-L-arginine methyl ester
NMR: Nuclear magnetic resonance
nNOS: neuronal NOS
NO: Nitric oxide
PKB: Protein kinase B
SBP: Systolic blood pressure
sGC: soluble guanylyl cyclase
SMC: Smooth muscle cells
TFA: Trifluoroacetic acid
TLC: Thin-layer chromatography
