# Supplementary material for: Lancemaside A, a major triterpene saponin of Codonopsis lanceolata enhances regulation of nitric oxide synthesis via eNOS activation
Source: BMC Complement Altern Med. 2019 May 24;19:110. doi: 10.1186/s12906-019-2516-6 (PMC6534936; doi:10.1186/s12906-019-2516-6)
Supplement: Supplementary file 1 — Figure S1. Determination of lancemaside A (LA) from C. lanceolata. (a) Scheme showing the isolation of LA from C. lanceolata. (b) Thin-layer chromatography, TLC analysis was performed to identify LA in the n-butanol-soluble portion (top panel). Open column was packed with the silica gel fractions (3–7) eluted by a dichloromethane-methanol solvent gradient (middle panel) and HPLC fractions (27–29) were separated by a linear gradient of solvent mixtures of 0.05% trifluoroacetic acid in H2O (TFA, solvent A) and acetonitrile (solvent B) (bottom panel) (DOCX 399 kb) [file 12906_2019_2516_MOESM1_ESM.docx]

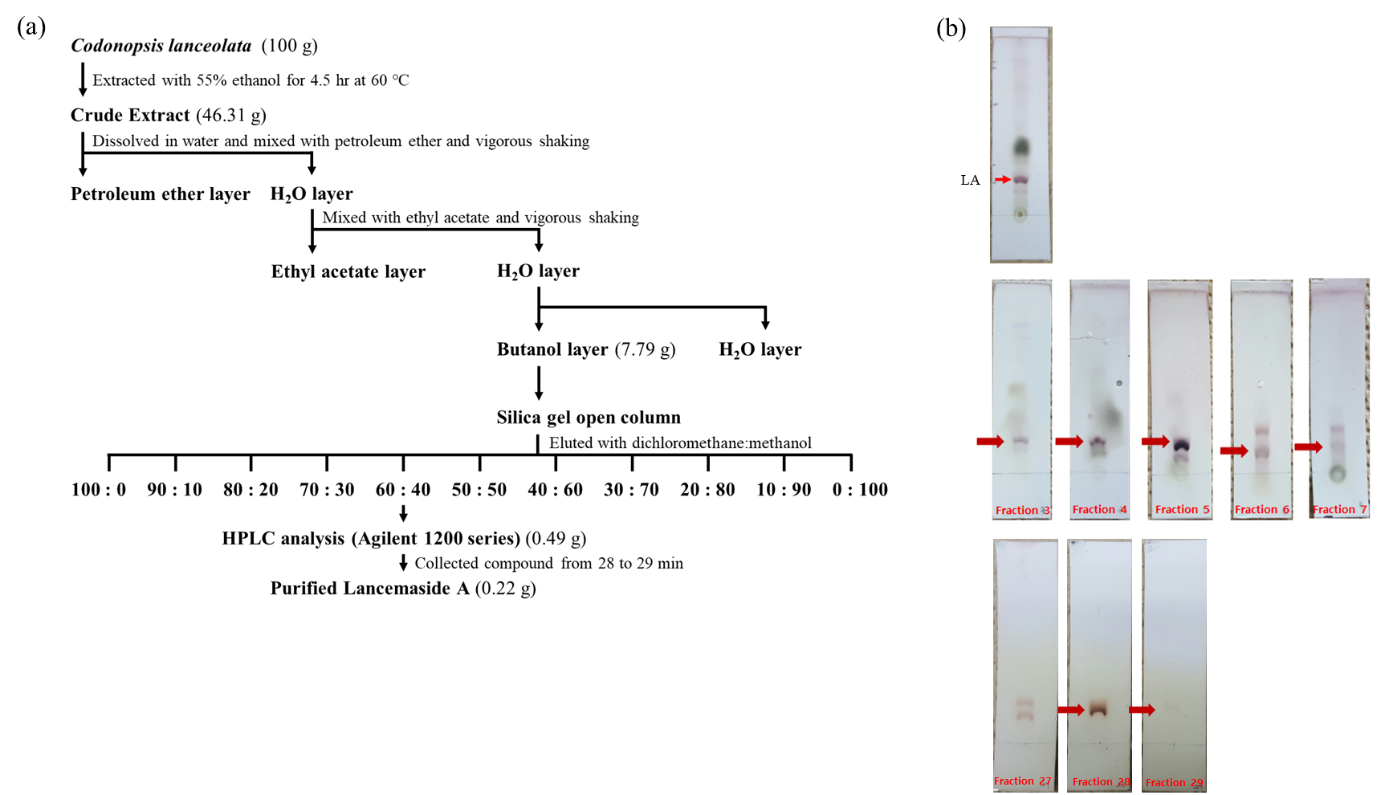


Additional file 1 **Figure S1. Determination of lancemaside A (LA) from *C. lanceolata*.** (a) Scheme showing the isolation of LA from *C. lanceolata.* (b) Thin-layer chromatography, TLC analysis was performed to identify LA in the *n*-butanol-soluble portion (top panel). Open column was packed with the silica gel fractions (3−7) eluted by a dichloromethane-methanol solvent gradient (middle panel) and HPLC fractions (27−29) were separated by a linear gradient of solvent mixtures of 0.05% trifluoroacetic acid in H_2_O (TFA, solvent A) and acetonitrile (solvent B) (bottom panel).
