# Supplementary material for: Lancemaside A, a major triterpene saponin of Codonopsis lanceolata enhances regulation of nitric oxide synthesis via eNOS activation
Source: BMC Complement Altern Med. 2019 May 24;19:110. doi: 10.1186/s12906-019-2516-6 (PMC6534936; doi:10.1186/s12906-019-2516-6)
Supplement: Supplementary file 2 — Table S1. Liquid chromatography-tandem mass spectrometry (LC-MS/MS) conditions for the determination of lancemaside A (LA) (A) and summary of MS & MS/MS chromatogram spectrum in negative ion mode of LA (B) (DOCX 25 kb) [file 12906_2019_2516_MOESM2_ESM.docx]

Additional file 2: **Table S1. Liquid chromatography-tandem mass spectrometry (LC-MS/MS) conditions for the determination of lancemaside A (LA) (A) and summary of MS & MS/MS chromatogram spectrum in negative ion mode of LA (B)**

(A)

| Instrument | LC : Nexera X2 (Shimadzu, Kyoto, Japan) MS/MS : LCMS-8050 (Shimadzu, Kyoto, Japan) | | |
| --- | --- | --- | --- |
| HPLC Conditions | | | |
| Column | ACQUITY BEH C_18_  (2.1 mm i.d. × 100 mm, 1.7 μm) | | |
| Solvent | A: DW (0.1 % Formic acid)  B: ACN (0.1 % Formic acid) | | |
| Elution Condition | Time (min) | A (%) | B (%) |
|  | 0 | 80 | 20 |
|  | 5 | 0 | 100 |
|  | 7 | 0 | 100 |
|  | 7.1 | 80 | 20 |
|  | 10 | 80 | 20 |
| Flow Rate | 0.3 mL/min | | |
| Injection Volume | 2 μL | | |
| MS/MS Conditions | | | |
| Detection Ion mode | ESI negative mode ([M-H]^-^) | | |
| Interface Voltage | 4.0 kV | | |
| Interface Temp. | 120^◦^C | | |
| Desolvation line Temp. | 250^◦^C | | |
| Neubulizing gas Flow | 3 L/min | | |
| Drying gas Flow | 10 L/min | | |

| Compound | R_t_^a^ (min) | Molecular weight | Formula | Exact mass (*m/z*) [M-H]^-^ |
| --- | --- | --- | --- | --- |
| Lancemaside A | 3.11 | 1190.6 | C_57_H_90_O_26_ | 1189.6 |
|  | Product mass (*m/z*) [M-H]^-^ | Q1 Pre Bias (V) | CE^b^ | Q3 Pre Bias (V) |
|  | 647.5^*^ | 34 | 55 | 22 |
|  | 469.2 | 34 | 55 | 22 |
|  | 585.3 | 34 | 50 | 20 |
| ^a^Retention time | |  |  |  |
| ^b^Collision energy (eV) | |  |  |  |
| ^*^Major fragmentation ion | |  |  |  |

(B)
